# Wild bird surveillance for highly pathogenic avian influenza H5 in North America

**DOI:** 10.1186/s12985-015-0377-2

**Published:** 2015-09-28

**Authors:** Paul L. Flint, John M. Pearce, J. Christian Franson, Dirk V. Derksen

**Affiliations:** U.S. Geological Survey, Alaska Science Center, Anchorage, AK USA; U.S. Geological Survey, National Wildlife Health Center, Madison, WI USA

## Abstract

It is unknown how the current Asian origin highly pathogenic avian influenza H5 viruses arrived, but these viruses are now poised to become endemic in North America. Wild birds harbor these viruses and have dispersed them at regional scales. What is unclear is how the viruses may be moving from the wild bird reservoir into poultry holdings. Active surveillance of live wild birds is likely the best way to determine the true distribution of these viruses. We also suggest that sampling be focused on regions with the greatest risk for poultry losses and attempt to define the mechanisms of transfer to enhance biosecurity. Responding to the recent outbreaks of highly pathogenic avian influenza in North America requires an efficient plan with clear objectives and potential management outcomes.

## Background

The original United States interagency strategic plan for detection of Asian highly pathogenic H5N1 avian influenza was developed nearly a decade ago, with focus on the early detection of an exotic virus [1]. The current outbreaks of HPAI H5N8 and H5N2, caused by foreign origin viruses, have very quickly become widely distributed within North America, thus negating the need for early detection at the broad scale. Two new avian influenza surveillance and monitoring documents have been recently released that address early detection at local and regional scales. One focuses on detection of HPAI in waterfowl in high priority watersheds and the spread of viruses to new areas of concern [2]. The other encompasses a strategy for early detection of avian influenza viruses of significance in wild birds in general, and encourages sampling of areas with high poultry density [3]. We propose taking these plans a step further by suggesting a 2-tiered surveillance strategy focusing on waterfowl and bridge species in the vicinity of poultry operations. This 2-step sampling design would address the mechanism(s) of virus transfer and provide data that can inform management actions that minimize the impact of these viruses on domestic poultry.

## Main text

It is unknown how HPAI H5 viruses from Asia reached North America [4–7]. Asian origin HPAI H5 viruses were first detected in North America in November of 2014 in poultry farms along the Fraser River in southwestern Canada but it is unknown how these viruses were introduced [8]. Extensive sampling for the Asian HPAI H5N1 from 2006-2010 failed to reveal evidence of intact foreign avian influenza (AI) viruses in North American wild birds [9]. However, numerous AI viruses isolated from wild birds in North America were shown to contain individual genes that originated in Asia leading to the conclusion that wild birds do transfer viruses among continents, although these viruses appear to be reassorted into the local AI community [10–12]. Thus, the detection in November of 2014 of a non-reassorted AI virus in North America [13] is unusual, although not unprecedented [14]. Further sampling in early 2015 confirmed that the Asian origin HPAI H5N8 subsequently reassorted with endemic viruses into at least two different HPAI subtypes, H5N2 and H5N1 [7, 8]. Thus, within a small geographic area and a short period of time, HPAI H5 viruses of foreign origin were identified in both poultry and wild birds in western North America. A key question involves the transmission pathway. Does this represent long distance-intercontinental dispersal of HPAI H5N8 by wild birds with subsequent transmission to poultry [6]? Alternatively, were the viruses introduced directly into poultry from an unknown source with subsequent dispersal into wild birds? Given the available data, both mechanisms are plausible and we cannot distinguish between these competing hypotheses.

Regardless of introductory mechanism, the HPAI H5 is now poised to become endemic in wild birds of North America [4], with the question of transmission pathway now more relevant at regional and local scales. Sampling within the Pacific Flyway of North America suggests that wild birds have dispersed HPAI viruses within this region [15]. Similarly, opportunistic sampling within the Mississippi Flyway demonstrates that HPAI viruses were more broadly distributed in space and time than detected in poultry outbreaks [15]. Equating the timing of detection in wild birds with the timing of arrival in a given area requires the assumption that sampling intensity was sufficient to detect the virus with high sensitivity (*i.e.*, probability of virus detection when present in the population under surveillance). Given that active surveillance has been opportunistic, the sensitivity of the sampling is unknown, but may have been low, resulting in uncertainty regarding timing of dispersal. Equating patterns of poultry outbreaks of a virus that originated in wild birds with actual patterns of virus distribution and spread requires even more assumptions.

### Movement of AI from wild birds to poultry

**Fig. 1:**
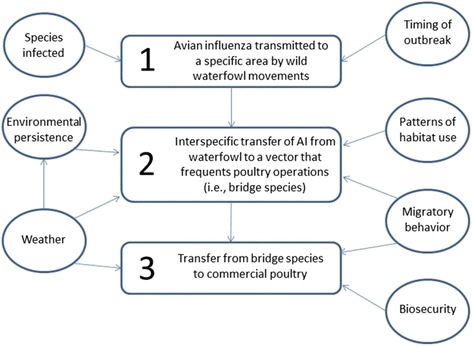
Conceptual model of the process of introduction and wild bird dispersal of HPAI and subsequent infection of commercial poultry holdings. The rectangular boxes (1-3) represent probabilities and the circles represent external factors that influence the probabilities. It is unlikely that direct transfer between waterfowl and poultry occurs. It has been proposed that “bridge species” that interact or share habitats with wild waterfowl and occur on poultry farms facilitate the transfer [14, 15]. Potential bridge species may be birds, rodents, and/or invertebrates

We developed a conceptual model that characterizes the process of AI transmission from wild birds into poultry holdings (Fig. 1). The widespread distribution of HPAI in wild birds in the Pacific Flyway (seven states) combined with the small number of reported commercial poultry outbreaks (two in CA) in the same area implies that completion of the pathway described in the model (*i.e.*, the product of all three probabilities) may be relatively rare. Given that this is a rare event, the time lag between transmission of a virus to a specific area by wild birds and transfer leading to infection of poultry is unknown. The full distribution of HPAI H5 viruses in wild birds in the Mississippi Flyway remains unknown, but the timeline where poultry outbreaks occurred from north to south at a time when general waterfowl migration was moving in the opposite direction may simply represent variable time lags between introduction of the virus and its appearance in poultry holdings. We hypothesize that environmental factors likely influence the process of AI transmission from wild birds into poultry. For example, severe weather may encourage bridge species to try and enter poultry barns for food and/or shelter. Alternatively, warm weather may increase access for bridge species as barns are opened for ventilation. If the probabilities in our model are influenced by environmental conditions, there is no basis for assuming a consistent time lag. Further there is likely spatial and temporal variation in the probabilities within our model. For example, the ratio of commercial poultry farms in the Mississippi to the Pacific Flyway is about 4:1 and the comparable ratio of HPAI H5 outbreaks is >50:1 [15]. Thus, it appears that the probability of completing the modeled process varied among regions or time periods. Little is known regarding the mechanisms of transfer between wild waterfowl and commercial poultry and external factors which may alter those mechanisms.

Because HPAI H5 viruses were found in waterfowl species in numerous locations in both the Pacific and Mississippi Flyways, we assumed that the first probability in Fig. 1 of our conceptual model was very high (*i.e.*, 1.0 at numerous locations). Relatively little is known about potential bridge species but they likely vary by region and in some cases by season. Burns et al. [16] identified 121 species of wild birds that occurred in the vicinity of commercial poultry operations in Canada and Caron et al. [17] described > 200 species from a series of sites in Zimbabwe. However, both studies identified a much smaller sub-set of species that were common and considered capable of transmitting AI. Specifically, Burns et al. [16] noted European Starlings (*Sturnus vulgaris*) and Rock Pigeons (*Columba livia*) as two wild bird species most likely to enter commercial poultry facilities. Boon et al. [18] inoculated starlings and pigeons with HPAI H5N1 and documented active infections with no mortality. Thus, we suggest that these two common and wide spread birds are examples of potential bridge species for HPAI in North America. Other studies have documented HPAI in insects [19–21] and LPAI in rodents [22] making the potential range of bridge species quite large.

### Effective wild bird surveillance

Detection of HPAI H5N8 and H5N2 in wild waterfowl in Asia, Europe, and North America has resulted from active surveillance and isolated dead bird investigations [13, 23, 24]. Knight-Jones et al. [25] conducted detailed analyses comparing various surveillance sampling techniques used to detect Asian HPAI H5N1 at Lake Constance in Europe. They concluded that sentinel bird sampling and testing of birds found dead were the most sensitive and cost effective techniques. However, they noted that the utility of dead bird testing is highly dependent on the probability of a dead bird being found and mortality rates. In their review, Hoye et al. [26] concluded that dead bird surveillance cannot be used to infer complete presence or absence distributions of HPAI if the viruses do not cause mortality. Kang et al. [27] exposed wild caught Mallards (*Anas platyrhynchos*) and Baikal Teal (*Anas formosa*) to HPAI H5N8 in a controlled trial and noted that all birds became infected and shed virus at high rates. No mortality occurred in 41 inoculated Mallards but 1 of 2 teal died without showing clinical signs and the cause of death was not determined. In a reported mortality event in South Korea, HPAI H5N8 was isolated from tissues of eight waterbird species and Baikal Teal, in particular, exhibited a combination of clinical signs and gross and microscopic lesions leading the authors to conclude that HPAI was a cause of mortality [28]. Thus, Baikal Teal may be susceptible to H5N8, but Jeong et al. [24] found H5 antibodies in >50 % of live/wild Baikal Teal sampled, implying that the realized mortality rate in the wild must be fairly low. Zhao et al. [29] exposed 6-week-old Mallards to HPAI H5N8 and similarly observed no mortality within 14 days. Given the apparent low mortality rate for wild birds exposed to HPAI H5N8 some authors have concluded that active surveillance is the preferred approach [23, 27, 30, 31]. Further, the apparent spread of HPAI H5N8 in wild birds in North America (from Washington south within the Pacific Flyway) has been primarily detected with active surveillance and is notable for a dearth of reported mortality events. Thus, active directed surveillance sampling of live birds appears to have the highest sensitivity for determining the true distribution of the HPAI H5 viruses currently circulating in wild birds in North America. Testing of dead birds associated with mortality investigations may provide an ancillary source of information on avian influenza viruses, revealing new geographic locations of H5 viruses or changes in viral pathogenicity or host range [2, 26]. However, such sampling is, by its very nature, opportunistic and unpredictable, limiting inference regarding the distribution of these viruses.

**Fig. 2:**
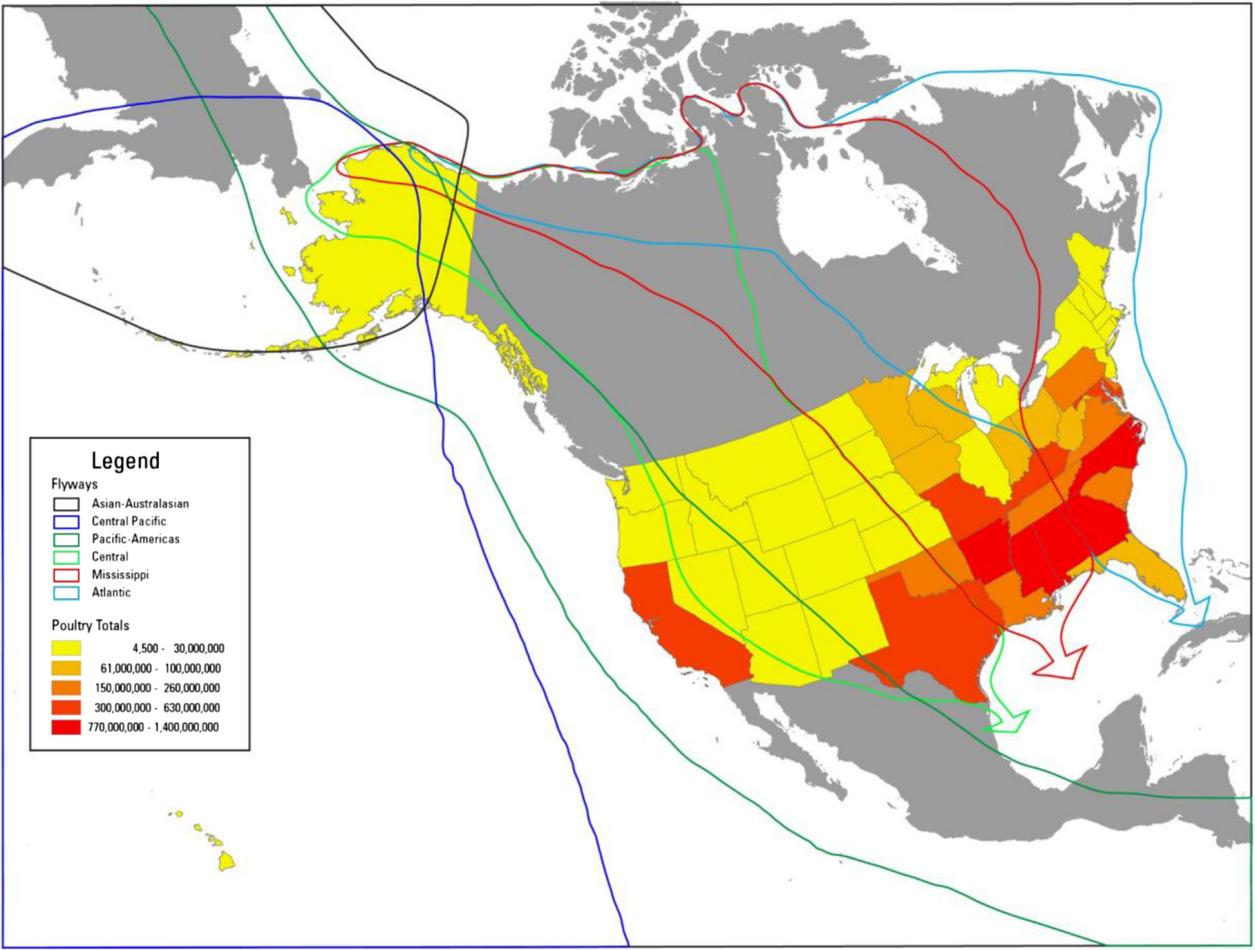
Poultry sales in 2012 by state in relation to major waterfowl flyways in North America. Poultry sales (layers, pullets, broilers, turkeys) as an index of density can be used to stratify wild bird surveillance sampling during the non-breeding season. This approach would target sampling in locations where the greatest risk of economic damage could occur. Sampling at high latitudes during the breeding season when populations from the various flyways (including Asian flyways) overlap can identify the potential for spread among the flyways. Poultry data from the 2012 Census of Agriculture [13]

As more is learned about the epizootiology of HPAI H5 viruses, surveillance techniques in North America and elsewhere can be tailored to optimize detection. Furthermore, the HPAI situation in North America presents an opportunity to investigate the potential movement of these viruses among wild birds and poultry facilities. At this time, the Centers for Disease Control and Prevention considers the risk posed to human health by the HPAI H5 viruses found in wild birds and poultry in North America to be low [32]. Yet, these viruses pose substantial risk to the commercial poultry industry where losses have significant economic impacts. Therefore, a tiered sampling approach in response to HPAI H5 viruses currently circulating in North America may be the most efficient surveillance strategy. Such an approach would allow sampling to be segregated seasonally and geographically, and targeted based on risk. Machalaba *et al.* [31] noted that high latitude sampling associated with breeding areas seem to indicate relative “hotspots” for AI. These high latitude areas also occur in regions where birds from multiple flyways overlap creating the potential for broad scale dispersal of viruses along an East-West gradient (Fig. 2) [7]. Thus, breeding season sampling would focus on higher latitude areas and assess the risk for spread across flyways. Wild bird surveillance sampling outside of the breeding season could incorporate a risk-based approach by focusing in areas with the highest density of poultry (Fig. 2), with stratification at multiple levels (flyway, state, and county). Retrospective studies have demonstrated that the likelihood of AI outbreaks in poultry increased with proximity to wetlands [32–34]. Thus, habitat mapping could be used to target sampling at farms with the greatest risk.

## Conclusions

Surveillance activities should go beyond simply documenting and tracking where HPAI H5 viruses occur, but rather target sampling in areas where management outcomes can be applied that enhance biosecurity. The existing HPAI surveillance plan for waterfowl focuses on early detection of viruses in specific areas (Fig. 1, box 1), selected on the basis of watershed characteristics, dabbling duck populations and movements, and previous AI activity [2]. For example, that plan calls for a greater sampling effort in areas of Utah and Nevada with prioritized watersheds and mixing of dabbling ducks but little commercial poultry production, than in the portions of Iowa and Minnesota where commercial poultry losses associated with H5N2 exceeded 40 million birds in spring 2015 [2, 15]. We propose that sampling be stratified in relation to the risk of economic damage to the poultry industry associated with these viruses by targeting areas of highest poultry density (Fig. 2) [3]. When HPAI is detected in a region, either in wild birds or poultry, directed sampling should immediately expand to include potential bridge species appropriate to specific locations and time (Fig. 1, box 2). Poultry facilities attract flies, rodents, and other pests and an investigation of 81 HPAI-positive turkey farms in the Midwestern U.S. in 2015 indicated that wild birds were observed within facilities on 35 % of farms [35, 36]. Wildlife surveys and poultry facility investigations can be used to identify high priority bridge species [16, 17,36]. Identification of the pathways by which HPAI can move from waterfowl into commercial poultry holdings can then be used to enhance and actively target biosecurity [31] (Fig. 1, box 3). We emphasize that in our conceptual model of HPAI movement, biosecurity is the only factor that could be controlled (Fig. 1). As such, a logical goal of a surveillance program would be to facilitate enhancement of biosecurity. Understanding which bridge species may be involved, when, how, and why they enter poultry facilities is necessary to define potential counter strategies. Clearly the response to avian vs mammalian bridge species would be different. It may also be productive to pursue identification of bridge species associated with low pathogenic AI infections, as these are common in wild waterfowl and occur annually in commercial poultry [37]. Detailed studies of epidemiological links between wild waterfowl and commercial poultry may provide a means to target biosecurity and lessen the economic impacts of the HPAI viruses currently circulating in in North America. Timely analyses of collected samples and rapid response to positive detections from surveillance results are also essential to facilitating management outcomes.

## Abbreviations

AI: Avian influenza
HPAI: Highly pathogenic avian influenza
CA: California
